# A Systematic Review and Meta-Analysis of Randomized Controlled Trials of Fecal Microbiota Transplantation for the Treatment of Inflammatory Bowel Disease

**DOI:** 10.1155/2022/8266793

**Published:** 2022-06-26

**Authors:** Xi-Yue Tan, Yu-Jia Xie, Xing-Long Liu, Xin-Yun Li, Bo Jia

**Affiliations:** College of Basic Medicine, Chengdu University of Traditional Chinese Medicine, Chengdu 611137, China

## Abstract

**Objectives:**

Inflammatory bowel disease (IBD) is a chronic recurrent inflammatory disease of the gastrointestinal tract, and its prevalence is increasing worldwide. Fecal microbiota transplantation (FMT) is an emerging therapy that modifies the patient's gut microbiota by transplanting feces from a healthy donor to achieve disease remission. However, its efficacy and safety need to be further investigated.

**Methods:**

PubMed, the Cochrane Library, Web of Science, Embase, and Google Scholar databases (up to 8th November 2021) were searched and literature was screened by title and abstract as well as full text. The primary outcome was clinical remission, with the clinical response as a secondary outcome. Risk ratios (RR) with 95% confidence intervals (CI) were reported.

**Results:**

A total of 14 trials were included in this study. In terms of clinical remission, FMT had a significant effect compared to placebo (RR = 1.44, 95 CI%: 1.03 to 2.02, *I*^2^ = 38%, *P*=0.03), with no significant risk of study heterogeneity. Moreover, FMT led to significant results in clinical response compared to placebo with moderate between-study heterogeneity (RR = 1.34, 95 CI%: 0.92 to 1.94, *I*^2^ = 51%, *P*=0.12). Subgroup analysis showed a higher clinical remission for fresh fecal FMT (40.9%) than that for frozen fecal FMT (32.2%); the efficacy of gastrointestinal (GI) pretreatment, the severity of disease, route of administration, and the donor selection remain unclear and require more extensive study. Safety analysis concluded that most adverse events were mild and self-resolving. The microbiological analysis found that the patient's gut microbiota varied in favor of the donor, with increased flora diversity and species richness.

**Conclusion:**

FMT is a safe, effective, and well-tolerated therapy. Studies have found that fresh fecal microbiota transplant can increase clinical remission rates. However, more randomized controlled trials and long-term follow-ups are needed to assess its long-term effectiveness and safety.

## 1. Introduction

Inflammatory bowel disease (IBD), which includes Crohn's disease (CD) and ulcerative colitis (UC), is a chronic recurrent inflammatory disease of the gastrointestinal tract [1]. The prevalence of IBD is increasing worldwide [2, 3]. The annual incidence of UC and CD in North America is similar, 0–19.2 per 100,000 and 0–20.2 per 100,000, respectively [2]. UC is characterized by diffuse mucosal inflammation, with lesions limited to the colonic mucosa and typical symptoms of diarrhea, mucus, and bleeding; CD can cause transmural inflammation that can accumulate anywhere in the gastrointestinal tract, most commonly in the terminal ileum or rectum, with abdominal pain, watery diarrhea, and weight loss as the main clinical features [1, 4, 5]. The etiology of IBD is uncertain, but it is now believed that genetic susceptibility, environmental exposure, gut microbiota, and the immune system are involved. Current treatment is aimed at controlling the progression of inflammation and achieving disease remission. Commonly used drugs are corticosteroids, antitumor necrosis factor-alpha (TNF-*α*) drugs, immunomodulators (e.g., methotrexate), biologic therapies, and surgery. IBD not only imposes a heavy financial burden and reduces the quality of life but also has a significant impact on the resources of the healthcare system [6, 7].

Fecal microbiota transplantation (FMT) is a therapy in which fecal matter from a healthy donor is infused into the gastrointestinal tract of a recipient patient, changing the patient's intestinal microbiota, leading to remission [8]. It has been suggested that the development of chronic inflammatory diseases such as IBD is associated with alterations in the composition of the intestinal microbiota (known as ecological dysbiosis) [9]. The differences in gut microbiota composition between IBD patients and healthy individuals are mainly in terms of microbial diversity and flora richness [10]. Extensive data confirm FMT as an effective treatment for recurrent *Clostridium difficile* infection (rCDI) [11, 12]. European Microbiology, the European Society for Infectious Diseases, and the American College of Gastroenterology all recommend FMT as a treatment for rCDI and have included it in their treatment guidelines [13, 14]. FMT is currently considered an emerging treatment for IBD [15, 16]. Studies have confirmed the efficacy of FMT in the treatment of IBD; Fang et al. [17] reported a clinical remission rate of 28.8% and a clinical response rate of 53% during follow-up in patients with IBD, concluding that fresh or frozen donor stool, route of administration, and antibiotic pretreatment or not had no effect on the efficacy of FMT for IBD. Caldeira et al. [18] showed an overall clinical remission rate of 37% and an overall clinical response rate of 54% in patients with IBD, suggesting that frozen fecal material was associated with a higher rate of clinical remission. However, several issues remain unresolved in the FMT protocol, such as type of stool (frozen or fresh), route of administration (lower or upper gastrointestinal tract), donor selection (healthy anonymous, or relative, or partners), and whether to pretreat the gastrointestinal (GI) tract (i.e., using vancomycin and/or polyethylene glycol, or antibiotic, or macrogol solution) prior to treatment with FMT.

Therefore, the purpose of this systematic review and meta-analysis is to synthesize data from published randomized controlled trials on FMT for the treatment of patients with IBD and to evaluate the efficacy and safety of FMT for the treatment of IBD.

## 2. Methods

This study was conducted and reported in accordance with the Preferred Reporting Items for Systematic Evaluation and Meta-Analysis (PRISMA (available (here))) guidelines.

### 2.1. Search Methodology

English articles of randomized controlled trials were searched in PubMed, the Cochrane Library, Web of Science, Embase, and Google Scholar databases (up to 8th November 2021). The databases were used (“Fecal Microbiota Transplantation” OR “Microbiota Transplantation, Fecal” OR “Transplantation, Fecal Microbiota” OR “Bacteriotherapy” OR “Intestinal Microbiota Transfer”) AND (“IBD” OR “inflammatory bowel disease” OR “Crohn (s) disease” OR “CD” OR “ulcerative colitis” OR “UC” OR “colitis”) for the keywords combinations and wildcards were thoroughly searched to ensure the completeness of the search results.

### 2.2. Inclusion and Exclusion Criteria

Studies of randomized controlled trials of patients with IBD treated with FMT were screened by a set of inclusion and exclusion criteria. Inclusion criteria were as follows: (1) patients with IBD aged > 18 years with no gender restriction; (2) randomized controlled trials; (3) studies with clearly described clinical endpoints; (4) journal articles; and (5) FMT by different modalities (i.e., nasogastric tube, nasoduodenal tube, colon/colonoscopy, enema, or oral) was allowed. Exclusion criteria were as follows: (1) in vitro or animal testing; (2) languages other than English or Chinese; (3) patients with concomitant *Clostridium difficile* infection (CDI) infection or other pathogens; (4) population noncompliance, e.g., pediatric IBD; (5) duplicate literature; (6) other types of literature: reviews, editorials, case reports, abstracts, meta-analysis, observational studies, surveys, and questionnaires; (7) no clinical results reported, or studies with incomplete data.

We went through the PRISMA flowchart to select studies for inclusion. One reviewer performed the initial screening based on titles and abstracts. Titles and abstracts of potentially eligible records were independently reviewed by a second reviewer. Identified studies were then independently screened in full by two reviewers. Final inclusion was determined by both reviewers based on inclusion and exclusion criteria. The senior author discussed and resolved any discrepancies in the selection process.

### 2.3. Data Extraction and Quality Assessment

Data were extracted and evaluated independently by two reviewers, recorded on self-designed forms, and any disagreements were discussed and resolved by a third reviewer. Extracted study data included (1) first author, study year, and site; (2) participant characteristics: total number of participants, number of controls, number of experiments, and disease severity; (3) type of intervention: type of stool (fresh or frozen), route of administration, relationship to donor, gastrointestinal (GI) pretreatment, IBD type, and follow-up time; (4) clinical outcomes: primary outcome (clinical remission) and secondary outcome (clinical response).

Study quality was assessed using the Cochrane Bias Tool and risks of bias included: selection bias, performance bias, detection bias, attrition bias, reporting bias, and other sources of bias.

### 2.4. Statistical Analysis

The efficacy of FMT for IBD will be assessed based on clinical remission or clinical response as defined by the respective study authors. Safety will be assessed by study-reported adverse events and serious adverse events. The analysis will be performed using Review Manager, version 5.1. Results were expressed as risk ratios (RR) and 95% confidence interval (CI). Mantel–Haenszel statistics (M-H) were used to represent the results. The heterogeneity of the study results was evaluated graphically using the inconsistency relative index (*I*^*2*^), and the *I*^2^ statistic (*I*^2^ ≥ as 50% using a random-effects model and *I*^2^ < 50% using a fixed-effects model) was used to evaluate statistical heterogeneity [19], and *P* value < 0.05 was considered statistically significant. In addition, subgroup analysis was performed according to disease severity, route of administration, type of stool storage (fresh/frozen), GI pretreatment, IBD type, gender, and donor-patient relationship. To assess the stability of the results, sensitivity analysis was performed using STATA 16.0 software. Potential publication bias was assessed using funnel plots and Egger's test.

## 3. Results

### 3.1. Literature Search

**Figure 1:**
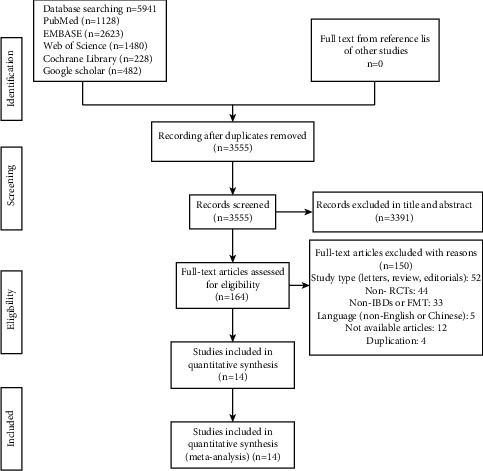
Selection flowchart.

A total of 5941 studies, including 5459 foreign language studies, and 482 Chinese language studies, were obtained by searching the database using the search strategy. The journal article title information of these studies was imported into the Endnote software. After removing duplicates, the search in the database yielded 3555 records. During the title and abstract screening process, 3391 records were considered irrelevant, and subsequently, 164 studies were evaluated after full-text assessment, and 150 studies were excluded using predetermined exclusion criteria. Ultimately, 14 studies were obtained, including 12 studies in English and 2 studies in Chinese. All included studies were published as journal articles. The study selection flowchart is shown in Figure 1. The data from these studies are presented in Table 1.

The 14 studies included 12 UC studies [20–23, 25, 26, 28–33] and 2 CD studies [24, 27] that included a total of 666 patients. FMT was the primary intervention in the RCTs trial group. In contrast, the control group took different measures, with 1 studying different routes of administration by gastroscopy or colonoscopy [27], 7 using placebo controls (including 2 autologous stools [20, 23], 2 sham procedures [24, 28], and 3 isotonic saline [21, 22, 25]), 1 using a special UC diet [30], 4 choosing conventional treatment as a control [29, 31–33], and 1 adding pectin to the FMT [26]. In 6 cases, the gastrointestinal (GI) tract was pretreated prior to FMT treatment (1 with antibiotic pretreatment [28], 4 with vancomycin and/or polyethylene glycol for bowel preparation [20, 23, 25, 26], and 1 with macrogol solution for rinsing the gastrointestinal tract [27]).

### 3.2. Bias Analysis of Included Studies

**Figure 2:**
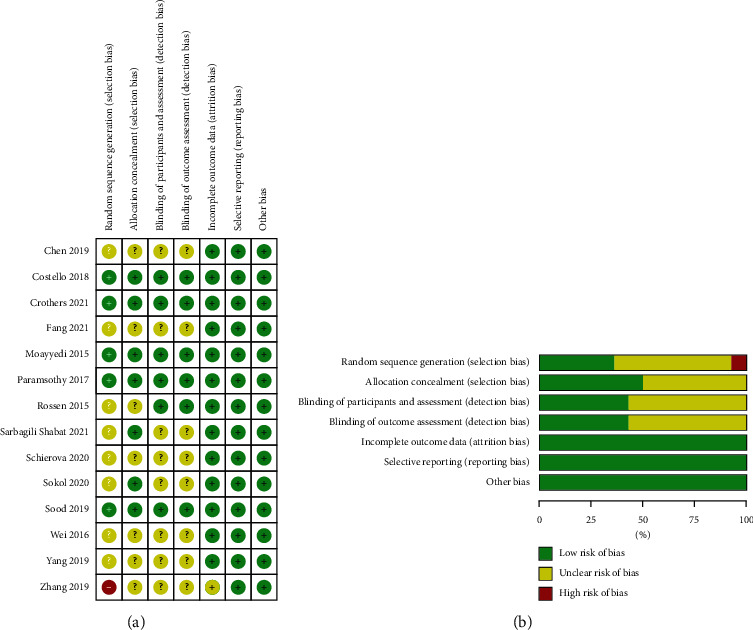
(a) Risk of bias summary of included RCTs. (b) Risk of bias graph of included RCTs.

Figures 2(a) and 2(b) summarizes the risk of bias for randomized controlled trials. The risk of bias assessment showed that 6 studies reported the method of randomization, of which 5 studies used computerized randomization as low risk [20–22, 25, 28], 1 study chose randomization according to the order of admission as high risk [33], and the remaining studies did not inform the specific grouping method. Seven studies took participant and researcher allocation concealment [20–22, 24, 25, 28, 30], 6 studies used participant and evaluator blinding [20–23, 25, 28], and the rest were not mentioned. Follow-up was not mentioned in only 1 study [33]. The remaining trials clearly reported the number of missed visits and dropouts and the reasons for them and adequately addressed the issue of selective reporting bias. However, given the poor methodological quality, we noted an unclear risk of bias in most of the included trials.

### 3.3. Clinical Remission/Response of FMT for IBD

**Figure 3:**
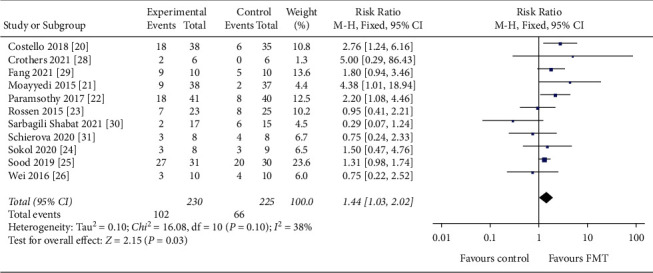
Forest plot of studies evaluating induction of clinical remission post-FMT for IBD. FMT, fecal microbiota transplantation; IBD, inflammatory bowel disease.

**Figure 4:**
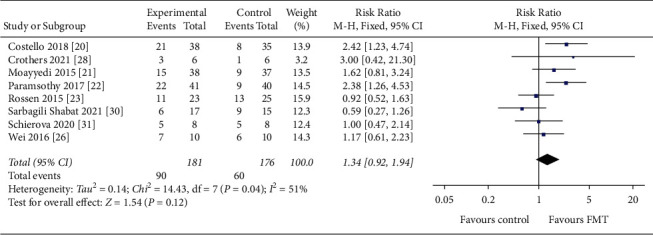
Forest plot of studies evaluating induction of clinical response post-FMT for IBD. FMT, fecal microbiota transplantation; IBD, inflammatory bowel disease.

Eleven studies reporting clinical remission in FMT for IBD (438 UC patients and 17 CD patients) were included in our analysis [20–26, 28–31]. In terms of clinical remission, FMT had a significant effect compared to placebo (RR = 1.44, 95 CI%: 1.03 to 2.02, *I*^*2*^ = 38%, *P*=0.03), with no significant risk of study heterogeneity. Eight studies reported clinical response [20–23, 26, 28, 30, 31], and FMT led to significant results in clinical response compared to placebo with moderate between-study heterogeneity (RR = 1.34, 95 CI%: 0.92 to 1.94, *I*^*2*^ = 51%, *P*=0.12). Forest plots are shown in Figures 3 and 4.

### 3.4. Subgroup Analysis

#### 3.4.1. Disease Severity

**Figure 5:**
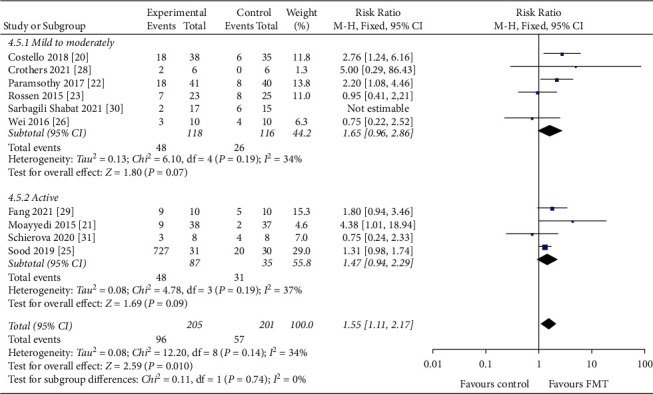
Forest plot of disease severity.

Of 455 patients, 266 were defined as having mild-moderate disease, of which 82 achieved clinical remission, and 172 were described as having active disease (including refractory UC and recurrent UC), of which 79 achieved clinical remission. The clinical remission rate for mild-moderate IBD was 30.8% (RR = 1.31, 95 CI%: 0.68 to 2.54, *I*^2^ = 56%), with moderate study heterogeneity. In comparison, the clinical remission rate for active IBD was 45.9% (RR = 1.47, 95 CI%: 0.94, 2.29, *I*^2^ = 37%), with a low risk of study heterogeneity. No statistically significant differences were observed between these two studies (*P*=0.14) (Figure 5).

#### 3.4.2. Type of Stool

**Figure 6:**
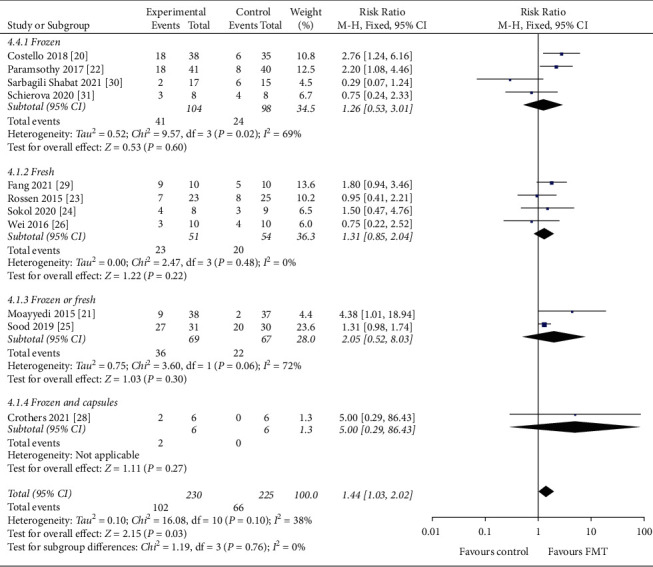
Forest plot of type of stool.

Of the 445 patients, 202 patients used frozen stools (65 patients achieved clinical remission), 105 patients used fresh stools (43 patients achieved clinical remission), 136 patients used frozen or fresh stools (58 patients achieved clinical remission), and 12 patients used frozen stools and oral capsules (2 patients achieved clinical remission). Clinical remission rates were 32.2% with frozen stool, 40.9% with fresh stool, 42.6% with frozen or fresh stool, and 16.7% with frozen stools and oral capsules (Figure 6). No statistically significant difference was observed between the type of stool groups for this analysis (*P*=0.10).

#### 3.4.3. Route of Administration

**Figure 7:**
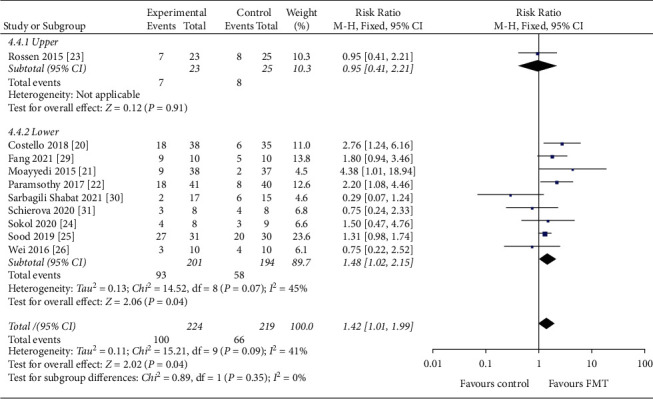
Forest plot of the route of administration.

Of the 443 patients, 395 patients were treated through the lower GI tract, 151 of whom achieved clinical remission, and 48 patients were treated through the upper GI tract, 15 of whom achieved clinical remission. The clinical remission rate was 38.2% for patients treated with the lower GI tract and 31.2% for those treated with the upper GI tract (Figure 7). No statistically significant differences were found between routes of administration in this analysis (*P*=0.09).

#### 3.4.4. Donor Selection

**Figure 8:**
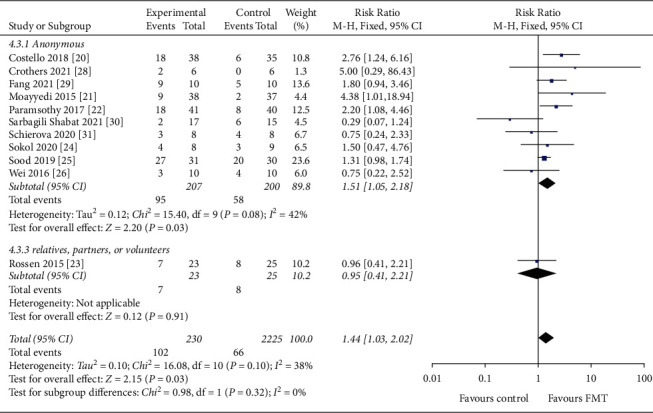
Forest plot of donor selection.

Of the 354 patients, 306 patients used stool from healthy anonymous donors, of whom 124 achieved clinical remission, and 48 patients used stool from relatives or close friends or volunteers, of whom 15 achieved clinical remission. The clinical remission rate was 37.6% with stools from healthy anonymous donors and 31.2% with stools from healthy relatives, partners, or volunteers (Figure 8). No statistically significant differences were found between these two studies (*P*=0.10).

#### 3.4.5. Gastrointestinal Pretreatment

**Figure 9:**
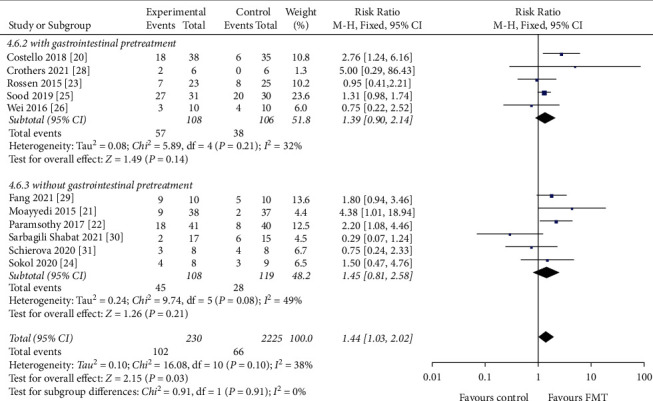
Forest plot of gastrointestinal pretreatment.

Of the 455 patients, 214 patients underwent GI pretreatment, of which 95 patients achieved clinical remission, and 241 patients did not undergo GI pretreatment, of which 73 patients achieved clinical remission. The clinical remission rate was 44.4% with GI pretreatment (RR = 1.39, 95 CI%: 0.90 to 2.14, *I*^2^ = 32%) and and 30.3% without GI pretreatment (RR = 1.45, 95 CI%: 0.81 to 2.58, *I*^2^ = 49%) (Figure 9). No statistically significant differences were found between these two studies (*P*=0.10).

#### 3.4.6. IBD Type

**Figure 10:**
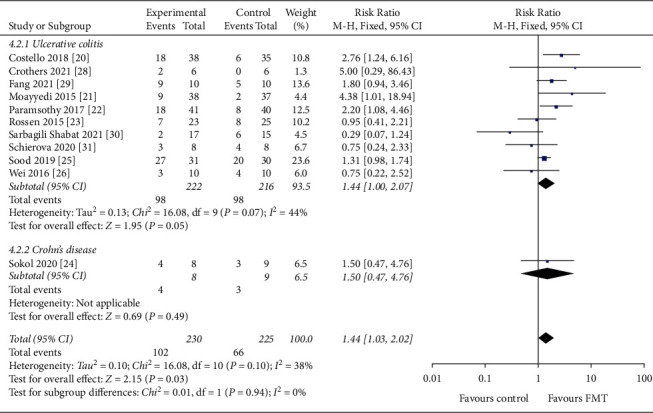
Forest plot of IBD type. IBD : inflammatory bowel disease.

Of the 455 patients, 438 were UC patients, of which 161 patients achieved clinical remission, and 17 were CD patients, of which 7 patients achieved clinical remission. The clinical remission rate was 36.8% had UC (RR = 1.44, 95 CI%: 1.00 to 2.07, *I*^2^ = 44%) and 41.2% had CD (RR = 1.50, 95 CI%: 0.47 to 4.76) (Figure 10). No statistically significant differences were found between these two studies (*P*=0.10).

#### 3.4.7. Gender

**Figure 11:**
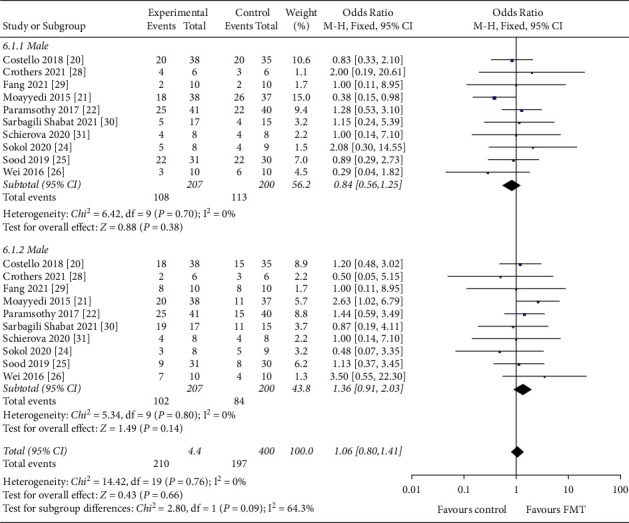
Forest plot of patients' gender.

Of the 407 patients, 221 were male and 186 were female. The percentage of males was 54.1% (RR = 0.84, 95 CI%: 0.56 to 1.25, *I*^*2*^ = 0%) and 45.7% (RR = 1.36, 95 CI%: 0.91 to 2.03) for females (Figure 11). No statistically significant differences were found between these two studies (*P*=0.76).

### 3.5. Safety and Adverse Events

Adverse events during and after treatment with FMT were reported in the vast majority of studies, with only one study not reporting follow-up. Self-limiting symptoms of gastrointestinal distress were the most common adverse events, including abdominal pain, nausea, diarrhea, abdominal distention, increased stool frequency, loss of appetite, and transient fever. These adverse events were mild and resolved on their own. Paramsothy et al. [22] found worsening or discomfort in two patients undergoing FMT, but the study found no significant association between individual donors or donor lots and adverse events. Therefore, consideration may not be related to FMT. Rossen et al. [23] reported four serious adverse events in their study (including two in the donor FMT group and two in the autologous FMT group), of which one patient was found to have small bowel CD, one required cervical cancer surgery, one was admitted with abdominal pain after 11 weeks of treatment that resolved on its own, and one became severely ill from a primo *Cytomegalovirus* infection 7 weeks after the first infusion and was found to be in the autologous FMT group, so no serious adverse events occurred related to donor FMT. No adverse events were considered to be associated with FMT. The duration of follow-up ranged from 4 weeks to 12 months.

### 3.6. Microbiological Analysis

Currently, for microbiological analysis, patient stool samples are examined by extracting bacterial DNA or high-throughput 16s rRNA gene sequencing to determine changes in patient stool flora before and after transplantation. FMT significantly changes the gut microbiota of IBD patients, increasing microbial diversity or abundance. Patients treated with donor's stool were found to have a more diverse gut microbiota in favor of the donor. A systematic review was conducted due to the lack of articles on microbiota analysis. Moayeddi et al. [21] found a statistically significant change in microbial composition with more diversity in the FMT group compared to the placebo group. The microbiota of the responders were more similar to those of the donor compared to the nonresponders but did not reach statistical significance. Sokol et al. [24] found that after excluding the two patients who failed FMT at week 6 (the microbiota of the donor was poorly colonized for these two patients), a significant difference was found between the FMT and placebo groups, and the similarity of the microbiota of the FMT group to the donor persisted until the end of follow-up. Crothers et al. [28] found that FMT did not increase Shannon diversity in the subjects but caused changes in gut microbiota levels, which were highly correlated with the donor after 2 weeks of FMT treatment and persisted until 20 weeks. This may be related to the antibiotic pretreatment of subjects prior to FMT treatment in this study. Fang et al. [29] reported that receiving single-donor FMT significantly reconstituted the intestinal microbiota and preserved documentation, with an increase in the proportion of Bacteroidetes and a decrease in the proportion of *Aspergillus*. The relative abundance of *Escherichia coli* in the gut of treated subjects decreased significantly; patients treated with FMT who achieved remission also tended to have higher Prevotella abundance.

### 3.7. Sensitivity Analysis Andpublicationbias

**Figure 12:**
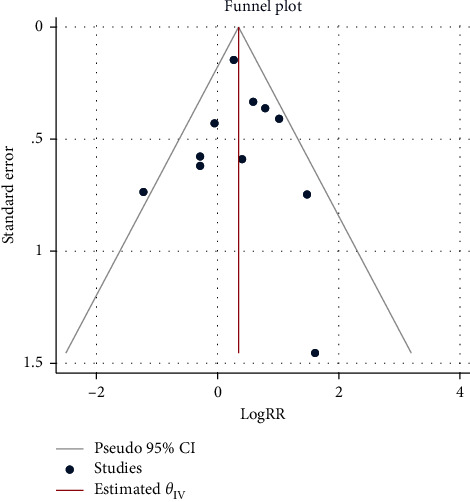
Funnel plots of publication bias. RR, risk ratio; CI, confidence interval.

Sensitivity analysis of clinical remission (primary outcome) showed that the effect sizes of the outcome indicators did not change significantly after the exclusion of any of the studies, suggesting that the results of the meta-analysis were more stable and credible. Funnel plots based on publication bias and Egger's test indicated that the conclusions were not affected by publication bias (*P*=0.714) (as shown in Figure 12).

## 4. Discussion

This article presents an updated systematic review and meta-analysis of randomized controlled trials of FMT for IBD. The overall study quality was low. Randomization, allocation concealment, and blinding of investigators or subjects or evaluators were the main factors that may have influenced the evaluation of bias, which explains the failure of randomized controlled trial studies to provide high-quality evidence. Most of the studies only mentioned “randomization” or “randomized grouping/assignment” in the text but did not specify the specific methods used and whether allocation concealment or blinding was implemented. In addition, these studies remained heterogeneous in design, with different treatment regimen designs (e.g., route of administration and choice of stool type) and different or poorly defined efficacy endpoints.

After FMT treatment, patients with IBD had a clinical remission rate of 36.9% (168/455) and a clinical response rate of 42.0% (150/357). Positive changes in clinical, endoscopic, or fecal microbiota were present. The clinical remission rate was 30.8% (82/266) for mild-moderate IBD and 45.9% (79/172) for active IBD. Our results suggest no significant difference in the degree of disease was found.

In our study, there was no significant difference found between the fresh stool (clinical remission rate: 40.9%) and frozen stool (clinical remission rate: 32.2%). Moreover, some studies have found that fresh FMT has superior clinical efficacy to frozen FMT [34]. Hamilton et al. [35] found that frozen stool microbiota from a healthy donor was more effective in treating recurrent CDI, thereby restoring the structure of the gut microbiota and clearing *C. difficile*. A randomized controlled trial [36] investigated the effectiveness of frozen FMT and fresh FMT in the treatment of recurrent CDI diarrhea, with clinical remission rates of 75.0% for frozen FMT and 70.3% for fresh FMT in a modified intention-to-treat analysis, showing similar efficacy and no major differences between the two stool types. The results of this study provide evidence for the use of fresh stools.

FMT can be administered via the upper GI tract (nasogastric tube, gastroduodenal tube, oral) or the lower GI tract (colonoscopy, cecum, enema), two different routes. A systematic review has shown that the lower GI tract is a safe and effective route for FMT in the treatment of CDI [37, 38]. In our study, the clinical remission rate was 38.2% (151/395) in the lower GI tract and 31.2% (15/48) in the upper GI tract. Our study suggests that no significant difference in the route of administration was found. Ramai et al. [39] concluded that colonoscopy has a better cure rate than the nasogastric tube for CDI. In addition to efficacy, patient compliance, cost-effectiveness, comfort of administration, level of invasiveness, risk of aspiration and infection, multiplicity of drugs to be administered, and relapse rate appear to be the main factors influencing physicians' choice of route of administration [40]. Some studies suggest that the use of upper GI administration requires attention to adverse events such as patient vomiting [41] and aspiration pneumonia [42], while there are also risks associated with lower GI administration in patients with severe colitis. Therefore, more extensive studies are needed to find low-risk, convenient, and cost-effective routes of administration.

At present, there is no consensus on the optimal donor for FMT. Our study found that fecal donors mainly included healthy adults, relatives or friends, or anonymous volunteers. Clinical remission was 37.6% (153/407) for feces from healthy anonymous donors and 31.2% (15/48) for feces from relatives, close friends, or volunteers. In our study, there was no significant difference found between the efficacy of FMT derived from healthy anonymous donors and the FMT using relatives, close friends, or volunteers. Some studies agree with our results that the ideal donor is a healthy donor [43]. Others have concluded that there is no significant difference between healthy donors and relative or close friend FMT [44]. The advantage of healthy anonymous donors is that they are easy to extract and screen from stool banks, saving time and money for screening stool donors [45]. Considering the possibility that the sample size using relatives or close friends was insufficient, more randomized controlled trials are needed to support our conclusions.

Clinical remission rates were 44.4% (95/214) and 30.3% (73/241) in the group with and without gastrointestinal pretreatment, respectively. In our study, there was no significant difference found between pretreatment of the gastrointestinal tract and treatment with FMT. One study found that bowel cleansing prior to colonoscopy and/or stool disposal led to changes in symptoms and gut microbiota composition in irritable bowel syndrome (IBS) [46]. Bowel cleansing brings immediate changes to the gut microbiota [47, 48], which plays an integral role in IBD. However, the amount of relevant trials is insufficient, so a more extensive study in this area is warranted. The clinical response rate was 36.8% (161/438) in UC patients and 41.2% (7/17) in CD patients. In our study, there was no significant difference found between CD patients and UC patients and the number of RCTs in CD is relatively insufficient, so more extensive research in this area is needed to support this conclusion. In our study, no significant differences were found in the gender of the patients.

Currently, the most common adverse events are self-limiting gastrointestinal discomfort, including abdominal pain, bloating, diarrhea, nausea, and other adverse events such as transient fever. These adverse events usually resolve on their own within 24 hours. The follow-up period is 6 weeks to 36 months.

The current systematic review still has some limitations. First, different infusion frequencies (single or multiple FMTs), number of donors, and preparation and storage of donor stools are factors that can affect the results, increasing heterogeneity between studies. Secondly, subgroup analysis (e.g., route of administration, type of stool, gastrointestinal pretreatment, and IBD type) has the limitation of a small number of studies and insufficient sample size. Finally, there is uncertainty regarding the selection of FMT donors, although some studies have shown no significant differences between healthy donors and relatives or close friends. Future larger and more extensive trials are needed to optimize the use of FMT, including donor selection, stool preparation and storage, and route of administration, to obtain better outcomes for patients.

## 5. Conclusions

In conclusion, our study found that FMT is an effective, feasible, and safe treatment for IBD. It has a positive effect in terms of clinical remission/clinical response. It can also be done by increasing the diversity and species richness of the gut microbiota. Still, further randomized controlled trials are needed to fully evaluate the long-term efficacy and safety of FMT in patients with IBD.

## Figures and Tables

**Table 1:** Data characteristics of the included studies.

Clinical response	Clinical remission	Follow-up	Study	Study type	Country	N	Severity	Donor	Route	Fresh/frozen
dFMT: 18/38 (47%); aFMT: 6/35 (17%), at week 8	dFMT: 21/38 (55%); aFMT: 8/35 (23%), at week 8	8 weeks	Costello et al. [20]	UC	Australia	73	Mild to moderate	Unrelated	Colonoscopy and enema (lower)	Frozen
FMT: 9 (24%); placebo: 2 (5%) at week 7	FMT: 15 (39%); placebo: 9 (24%) at week 7	6 weeks	Moayyedi et al. [21]	UC	Canada	75	Active	Unrelated	Retention enema (lower)	Fresh or frozen
FMT: 18 (44%); placebo: 8 (20%) at week 8	FMT: 22 (54%); placebo: 9 (23%) at week 8	8 weeks	Paramsothy et al. [22]	UC	Australia	81	Mild to moderate	Unrelated	Colonoscopy and enema (lower)	Frozen
dFMT: 6/23 (26.1%); aFMT: 8/25 (32%), at week 6; dFMT: 7/23 (30.4%); aFMT: 8/25 (32%), at week 12	dFMT: 10/23 (43.5%); aFMT: 13/25 (52.0%), at week 6; dFMT: 11/23 (47.8%); aFMT: 13/25 (52%), at week 12	12 weeks	Rossen et al. [23]	UC	Netherlands	48	Mild to moderate	Relatives, partners, or volunteers	Nasoduodenal tube (upper)	Fresh
FMT: 7/8 (87.5%); sham FMT: 4/9 (44.4%), at week 10; FMT: 4/8 (50.0%); sham FMT: 3/9 (33.3%), at week 24	—	12 weeks	Sokol et al. [24]	CD	France	17	Active	Unrelated	Colonoscopy (lower)	Fresh
FMT: 27/31 (87.1%); placebo: 20/30 (66.7%) at week 48	—	48 weeks	Sood et al. [25]	UC	India	61	Active	Unrelated	Colonoscopy infusion and enema (lower)	Fresh or frozen
FMT: 3/10 (30%); FMTP: 4/10 (40%) at week 12	FMT: 7/10 (70%); FMTP: 6/10 (60%) at week 12	12 weeks	Wei et al. [26]	UC	China	20	Mild to moderate	Unrelated	Colonoscopy (lower)	Fresh
Gastroscopy: 10/13 (76.9%); colonoscopy: 11/14 (78.6%) at week 2	Gastroscopy: 9/13 (69.2%); colonoscopy: 9/14 (64.3%) at week 2	8 weeks	Yang et al. [27]	CD	China	27	Active	Relatives, partners, or volunteers	Gastroscopy or colonoscopy (upper or lower)	Fresh
cFMT: 2/6 (33%); placebo: 0/6 (0%) at week 12	cFMT: 3/6 (50%); placebo: 1/6 (17%) at week 12	36 weeks	Crothers et al. [28]	UC	USA	17	Mild to moderate	Unrelated	Colonoscopy infusion and oral (lower and upper)	Frozen and capsules
FMT: 9/10 (90%); control: 5/10 (50%) at week 8	—	6–38 months	Fang et al. [29]	UC	China	20	Active	Unrelated	Colonoscopy (lower)	Fresh
FMT: 2/17 (11.8%); FMT + UCED: 4/19 (21.1%); UCED: 6/15 (40.0%) at week 8	FMT: 6/17 (35.3%); FMT + UCED: 8/19 (42.1%); UCED: 9/15 (60.0%) at week 8	12 weeks	Sarbagili Shabat et al. [30]	UC	Israel	51	Mild to moderate	Unrelated	Colonoscopy and enema (lower)	Frozen
FMT: 3/8 (37.5%); 5-ASA: 4/8 (50%) at week 12	FMT: 5/8 (62.5%); 5-ASA: 5/8 (62.5%) at week 12	12 weeks	Schierová et al. [31]	UC	Czech republic	16	Active	Unrelated	Enema (lower)	Frozen
FMT: 20/30 (66.67%); control: 12/30 (40.00%) at week 4	—	3 months	Chen et al. [32]	UC	China	60	NR	Unrelated	Enema (lower)	Fresh or frozen
FMT: 48/50 (96%); 5-ASA: 35/50 (70%) at week 2	—	NR	Zhang et al. [33]	UC	China	100	NR	Relatives, partners	Enema (lower)	NR

UC, ulcerative colitis; CD, Crohn's disease; N, number of patients; aFMT, autologous FMT; dFMT, donor FMT; cFMT, capsules FMT; FMTP, FMT combined with pectin; FMT, fecal microbiota transplantation; 5-ASA, 5-aminosalicylic acid; UCED, ulcerative colitis exclusion diet; NR, not reported.

## Data Availability

The data supporting this meta-analysis are from previously reported studies and datasets, which have been cited. The processed data are available from the corresponding author upon request.

## Supplementary Materials

Supplementary Materials See PRISMA_2020_checklist in the Supplementary Materials for detailed Preferred Reporting Items for Systematic Reviews and Meta-Analyses (PRISMA).

## References

[1] Xavier R. J., Podolsky D. K. (2007). Unravelling the pathogenesis of inflammatory bowel disease. *Nature*.

[2] Ananthakrishnan A. N. (2015). Epidemiology and risk factors for IBD. *Nature Reviews Gastroenterology & Hepatology*.

[3] Malik T. A. (2015). Inflammatory bowel disease: historical perspective, epidemiology, and risk factors. *Surgical Clinics of North America*.

[4] Zhang Y. Z., Li Y. Y. (2014). Inflammatory bowel disease: pathogenesis. *World Journal of Gastroenterology*.

[5] Flynn S., Eisenstein S. (2019). Inflammatory bowel disease presentation and diagnosis. *Surgical Clinics of North America*.

[6] Lessa F. C., Mu Y., Bamberg W. M. (2015). Burden of *Clostridium difficile* infection in the United States. *New England Journal of Medicine*.

[7] McGlone S. M., Bailey R. R., Zimmer S. M. (2012). The economic burden of *Clostridium difficile*. *Clinical Microbiology and Infection: the Official Publication of the European Society of Clinical Microbiology and Infectious Diseases*.

[8] Wang J. W., Kuo C. H., Kuo F. C. (2019). Fecal microbiota transplantation: review and update. *Journal of the Formosan Medical Association*.

[9] Kim D., Zeng M. Y., Nunez G. (2017). The interplay between host immune cells and gut microbiota in chronic inflammatory diseases. *Experimental & Molecular Medicine*.

[10] Cuppens T., Annibali D., Coosemans A. (2017). Potential targets’ analysis reveals dual PI3K/mTOR pathway inhibition as a promising therapeutic strategy for uterine leiomyosarcomas - an ENITEC group initiative. *Clinical Cancer Research*.

[11] Drekonja D., Reich J., Gezahegn S. (2015). Fecal microbiota transplantation for *Clostridium difficile* infection: a systematic review. *Annals of Internal Medicine*.

[12] Kassam Z., Lee C. H., Yuan Y., Hunt R. H. (2013). Fecal microbiota transplantation for *Clostridium difficile* infection: systematic review and meta-analysis. *American Journal of Gastroenterology*.

[13] Surawicz C. M., Brandt L. J., Binion D. G. (2013). Guidelines for diagnosis, treatment, and prevention of *Clostridium difficile* infections. *American Journal of Gastroenterology*.

[14] Debast S. B., Bauer M. P., Kuijper E. J. (2014). European society of clinical microbiology and infectious diseases: update of the treatment guidance document for *Clostridium difficile* infection. *Clinical Microbiology and Infection: the Official Publication of the European Society of Clinical Microbiology and Infectious Diseases*.

[15] Weisshof R., El Jurdi K., Zmeter N., Rubin D. T. (2018). Emerging therapies for inflammatory bowel disease. *Advances in Therapy*.

[16] Weingarden A. R., Vaughn B. P. (2017). Intestinal microbiota, fecal microbiota transplantation, and inflammatory bowel disease. *Gut Microbes*.

[17] Fang H., Fu L., Wang J. (2018). Protocol for fecal microbiota transplantation in inflammatory bowel disease: a systematic review and meta-analysis. *BioMed Research International*.

[18] Caldeira L. D. F., Borba H. H., Tonin F. S., Wiens A., Fernandez-Llimos F., Pontarolo R. (2020). Fecal microbiota transplantation in inflammatory bowel disease patients: a systematic review and meta-analysis. *PLoS One*.

[19] Huedo-Medina T. B., Sanchez-Meca J., Marin-Martinez F., Botella J. (2006). Assessing heterogeneity in meta-analysis: Q statistic or I2 index?. *Psychological Methods*.

[20] Costello S. P., Hughes P. A., Waters O. (2019). Effect of fecal microbiota transplantation on 8-week remission in patients with ulcerative colitis: a randomized clinical trial. *JAMA*.

[21] Moayyedi P., Surette M. G., Kim P. T. (2015). Fecal microbiota transplantation induces remission in patients with active ulcerative colitis in a randomized controlled trial. *Gastroenterology*.

[22] Paramsothy S., Kamm M. A., Kaakoush N. O. (2017). Multidonor intensive faecal microbiota transplantation for active ulcerative colitis: a randomised placebo-controlled trial. *The Lancet*.

[23] Rossen N. G., Fuentes S., van der Spek M. J. (2015). Findings from a randomized controlled trial of fecal transplantation for patients with ulcerative colitis. *Gastroenterology*.

[24] Sokol H., Landman C., Seksik P. (2020). Fecal microbiota transplantation to maintain remission in Crohn’s disease: a pilot randomized controlled study. *Microbiome*.

[25] Sood A., Mahajan R., Singh A. (2019). Role of faecal microbiota transplantation for maintenance of remission in patients with ulcerative colitis: a pilot study. *Journal of Crohn’s and Colitis*.

[26] Wei Y., Gong J., Zhu W. (2016). Pectin enhances the effect of fecal microbiota transplantation in ulcerative colitis by delaying the loss of diversity of gut flora. *BMC Microbiology*.

[27] Yang Z., Bu C., Yuan W. (2020). Fecal microbiota transplant via endoscopic delivering through small intestine and colon: no difference for Crohn’s disease. *Digestive Diseases and Sciences*.

[28] Crothers J. W., Chu N. D., Nguyen L. T. T. (2021). Daily, oral FMT for long-term maintenance therapy in ulcerative colitis: results of a single-center, prospective, randomized pilot study. *BMC Gastroenterology*.

[29] Fang H., Fu L., Li X. (2021). Long-term efficacy and safety of monotherapy with a single fresh fecal microbiota transplant for recurrent active ulcerative colitis: a prospective randomized pilot study. *Microbial Cell Factories*.

[30] Sarbagili Shabat C., Scaldaferri F., Zittan E. (2021). Use of fecal transplantation with a novel diet for mild to moderate active ulcerative colitis: the CRAFT UC randomized controlled trial. *Journal of Crohn’s & Colitis*.

[31] Schierova D., Brezina J., Mrazek J. (2020). Gut microbiome changes in patients with active left-sided ulcerative colitis after fecal microbiome transplantation and topical 5-aminosalicylic acid therapy. *Cells*.

[32] Chen F.-Y., Zou K. (2019). Clinical efficacy of fecal bacteria transplantation in the treatment of ulcerative colitis. *Clinical Education of General Practice*.

[33] Zhang K.-Q., Jiang Q., Zhang H.-B. (2019). Effect of fecal microbiota transplantation on gastrointestinal function and intestinal flora in patients with ulcerative colitis. *World Chinese Journal of Digestology*.

[34] Agarwal A., Maheshwari A., Verma S. (2021). Superiority of higher-volume fresh feces compared to lower-volume frozen feces in fecal microbiota transplantation for recurrent clostridioides difficile colitis. *Digestive Diseases and Sciences*.

[35] Hamilton M. J., Weingarden A. R., Unno T., Khoruts A., Sadowsky M. J. (2013). High-throughput DNA sequence analysis reveals stable engraftment of gut microbiota following transplantation of previously frozen fecal bacteria. *Gut Microbes*.

[36] Lee C. H., Steiner T., Petrof E. O. (2016). Frozen vs fresh fecal microbiota transplantation and clinical resolution of diarrhea in patients with recurrent *Clostridium difficile* infection: a randomized clinical trial. *JAMA*.

[37] Cohen N. A., Livovsky D. M., Yaakobovitch S. (2016). A retrospective comparison of fecal microbial transplantation methods for recurrent *Clostridium difficile* infection. *The Israel Medical Association Journal*.

[38] Furuya-Kanamori L., Doi S. A., Paterson D. L. (2017). Upper versus lower gastrointestinal delivery for transplantation of fecal microbiota in recurrent or refractory *Clostridium difficile* infection: a collaborative analysis of individual patient data from 14 studies. *Journal of Clinical Gastroenterology*.

[39] Ramai D., Zakhia K., Fields P. J. (2021). Fecal microbiota transplantation (FMT) with colonoscopy is superior to enema and nasogastric tube while comparable to capsule for the treatment of recurrent *Clostridioides difficile* infection: a systematic review and meta-analysis. *Digestive Diseases and Sciences*.

[40] Gulati M., Singh S. K., Corrie L., Kaur I. P., Chandwani L. (2020). Delivery routes for faecal microbiota transplants: available, anticipated and aspired. *Pharmacological Research*.

[41] Gweon T. G., Kim J., Lim C. H. (2016). Fecal microbiota transplantation using upper gastrointestinal tract for the treatment of refractory or severe complicated *Clostridium difficile* infection in elderly patients in poor medical condition: the first study in an Asian country. *Gastroenterology Research and Practice*.

[42] Vermeire S., Joossens M., Verbeke K. (2016). Donor species richness determines faecal microbiota transplantation success in inflammatory bowel disease. *Journal of Crohn’s and Colitis*.

[43] Bibbo S., Settanni C. R., Porcari S. (2020). Fecal microbiota transplantation: screening and selection to choose the optimal donor. *Journal of Clinical Medicine*.

[44] Jung Lee W., Lattimer L. D. N., Stephen S., Borum M. L., Doman D. B. (2015). Fecal microbiota transplantation: a review of emerging indications beyond relapsing *Clostridium difficile* toxin colitis. *Gastroenterology and Hepatology*.

[45] Ramai D., Zakhia K., Ofosu A., Ofori E., Reddy M. (2019). Fecal microbiota transplantation: donor relation, fresh or frozen, delivery methods, cost-effectiveness. *Annals of Gastroenterology*.

[46] Holster S., Lindqvist C. M., Repsilber D. (2019). The effect of allogenic versus autologous fecal microbiota transfer on symptoms, visceral perception and fecal and mucosal microbiota in irritable bowel syndrome: a randomized controlled study. *Clinical and Translational Gastroenterology*.

[47] Drago L., Toscano M., de Grandi R., Casini V., Pace F. (2016). Persisting changes of intestinal microbiota after bowel lavage and colonoscopy. *European Journal of Gastroenterology and Hepatology*.

[48] Jalanka J., Salonen A., Salojarvi J. (2015). Effects of bowel cleansing on the intestinal microbiota. *Gut*.

